# Prediction of the Extent of Blood–Brain Barrier Transport Using Machine Learning and Integration into the LeiCNS-PK3.0 Model

**DOI:** 10.1007/s11095-025-03828-0

**Published:** 2025-02-10

**Authors:** Berfin Gülave, Helle W. van den Maagdenberg, Luke van Boven, Gerard J. P. van Westen, Elizabeth C. M. de Lange, J. G. Coen van Hasselt

**Affiliations:** 1 Systems Pharmacology and Pharmacy, Leiden Academic Center for Drug Research, Leiden University, Einsteinweg 55, 2333 CC Leiden, The Netherlands; 2 Medicinal Chemistry, Leiden Academic Center for Drug Research, Leiden University, Einsteinweg 55, 2333 CC Leiden, The Netherlands

**Keywords:** K_p,uu,BBB_, LeiCNS-PK3.0, machine learning, random forest

## Abstract

**Introduction:**

The unbound brain-to-plasma partition coefficient (K_p,uu,BBB_) is an essential parameter for predicting central nervous system (CNS) drug disposition using physiologically-based pharmacokinetic (PBPK) modeling. K_p,uu,BBB_ values for specific compounds are however often unavailable, and are moreover time consuming to obtain experimentally. The aim of this study was to develop a quantitative structure–property relationship (QSPR) model to predict the K_p,uu,BBB_ and to demonstrate how QSPR-model predictions can be integrated into a physiologically-based pharmacokinetic model for the CNS.

**Methods:**

Rat K_p,uu,BBB_ values were obtained for 98 compounds from literature or in house historical data. For all compounds, 2D and 3D physico-chemical and structural properties were derived using the Molecular Operating Environment (MOE) software. Multiple machine learning (ML) regression models were compared for prediction of the K_p,uu,BBB_, including random forest, support vector machines, K-nearest neighbors, and (sparse-) partial least squares. Finally, we demonstrate how the developed QSPR model predictions can be integrated into a CNS PBPK modeling workflow.

**Results:**

Among all ML algorithms, a random forest showed the best predictive performance for K_p,uu,BBB_ on test data with R^2^ value of 0.61 and 61% of all predictions were within twofold error. The obtained K_p,uu,BBB_ were successfully integrated into the LeiCNS-PK3.0 CNS PBPK model.

**Conclusions:**

The developed random forest QSPR model for K_p,uu,BBB_ prediction was found to have adequate performance, and can support drug discovery and development of novel investigational drugs targeting the CNS in conjunction with CNS PBPK modeling.

**Supplementary Information:**

The online version contains supplementary material available at 10.1007/s11095-025-03828-0.

## Introduction

Central nervous system (CNS) active drugs have to cross the blood–brain barrier (BBB) to exert their effect. This barrier may have an important impact on CNS drug distribution, which is one of the reasons contributing to the high attrition rate in CNS drug development [1]. Therefore, it is important to understand the CNS penetration of drugs. In this context, the ratio of the unbound drug concentrations in brain extracellular fluid (brain_ECF_) over plasma at steady state, or the K_p,uu,BBB_, is an essential parameter to predict CNS drug disposition. It is important to distinguish between K_p,uu,BBB_ and K_p,uu,brain_, as the latter is based on unbound concentrations of the brain, to which both extra- and intracellular concentrations contribute [2], so less specifically reflecting BBB transport. The K_p,uu,BBB_ is an indicator regarding the dominant transport mechanism of the compound, where a value around 1 indicates mainly passive transport, while K_p,uu,BBB_ values smaller or larger than 1 indicate dominant active efflux or influx BBB transport processes, respectively.

To determine the K_p,uu,BBB_, various techniques have been proposed. *In vivo* microdialysis is considered the gold standard due to its direct *in vivo* measurement of unbound concentration–time profiles in brain extracellular fluid (brain_ECF_) and in plasma, to determine clearance of the unbound drug into (Cl_in_) and clearance of the unbound drug out of the brain_ECF_ (Cl_out_) to determine K_p,uu,BBB_ in the full physiological setting [2–4]. Though being the best, it is not the easiest technique to be used, and also is restricted to drugs with not too high lipophilicity. Another approach to determine the K_p,uu,brain_, is the CMA approach [5] that utilizes an *in vivo* neuro PK study with total brain and total plasma concentrations to first establish the K_p,brain_. Next, the fraction unbound in plasma (f_u,plasma_) and fraction unbound in brain (f_u,brain_) are determined *in vitro*, and used to calculate K_p,uu,brain_. Using the volume of distribution in brain (V_u,brain_) instead of f_u,brain_ results in an estimation of K_p,uu,BBB_. So, in this approach, for determining K_p,uu,BBB_, a number of parameters are obtained outside the intact physiological context. The full physiological context in which microdialysis-derived K_p,uu,BBB_ values are obtained, makes such K_p,uu,BBB_ values more physiologically relevant.

The K_p,uu,BBB_ is a key parameter to enable the prediction of CNS pharmacokinetic profiles using CNS physiologically-based pharmacokinetic (PBPK) modelling. CNS PBPK modelling is a relevant computational approach to predict (unbound) drug distribution in the CNS, and to translate expected CNS drug disposition between species. We have previously developed and validated the LeiCNS-PK3.0 CNS PBPK model to predict unbound drug concentration–time profiles in multiple physiological compartments of the CNS [6]. However, CNS PBPK models require K_p,uu,BBB_ values to be provided as input parameters. To this end, QSPR models are relevant to generate K_p,uu,BBB_ values as input to the LeiCNS-PK3.0 model, when these are not available from experimental sources.

Although various *in vitro* and *in vivo* methods for K_p,uu,BBB_ determination are available, for the majority of existing drugs as well as for novel investigational compounds, K_p,uu,BBB_ values are unavailable. Quantitative structure–property relationship (QSPR) models link structural features of molecules with the pharmacological properties, and can be used to predict K_p,uu,BBB_. Several QSPR models have been developed to predict the total concentration of drug in the brain, based on total concentration in the blood at equilibrium (logBB) [7]. However, total concentration based models lack pharmacological relevance as these do not include the unbound concentration in brain_ECF_, which is responsible for the pharmacological effect. QSPR models using various machine learning algorithms which predict K_p,uu,BBB_, and do consider unbound drug concentrations in the brain_ECF_, based on data produced using the CMA approach have been previously described [8–15]. In addition, two QSPR models for K_p,uu,BBB_ have combined data generated using both *in vivo* microdialysis and the CMA approach [9, 16]. Finally, a QSPR model [16] was previously developed for datasets with K_p,uu,BBB_ values obtained by *in vivo* microdialysis study alone. However, while very elegant, this study used a relatively small sample size while more values are available. Most existing QSPR models for predicting K_p,uu,BBB_ are based on CMA derived values, complicating interspecies translatability. The scarcity of models utilizing microdialysis data, despite their higher physiological relevance, point out the need for QSPR models that prioritize microdialysis determined K_p,uu,BBB_ values.

The aim of this study was to develop a rat QSPR model to predict the K_p,uu,BBB_ values based on 98 *in vivo* microdialysis-derived K_p,uu,BBB_ values, and to compare the predictive performance for multiple machine learning algorithms. Moreover we aimed to demonstrate how the developed QSPR model can be integrated into the LeiCNS-PK3.0 CNS PBPK model.

## Methods

**Fig. 1:**
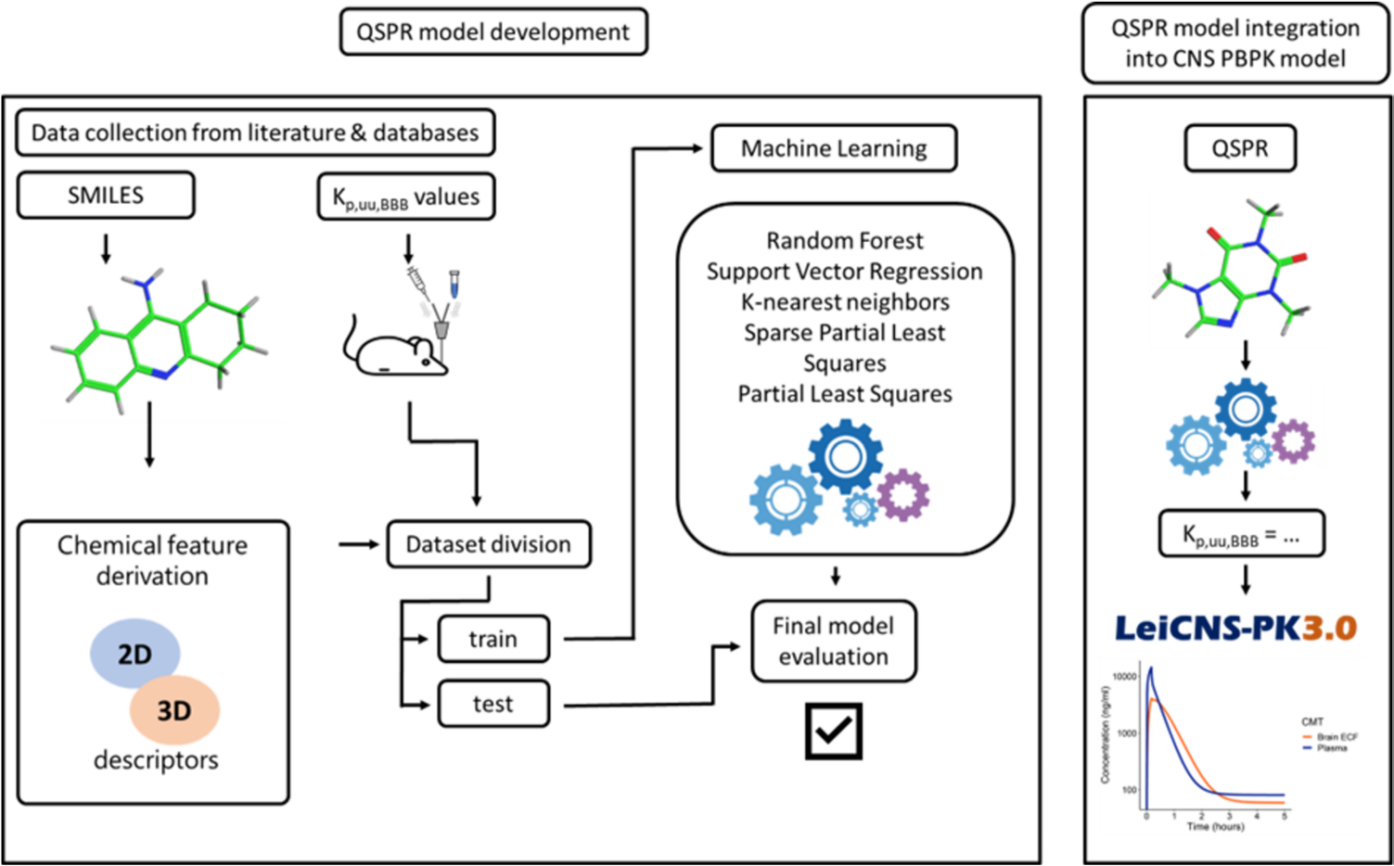
Overview of the quantitative structure–property relationship (QSPR) model development and application. Left panel shows the steps taken to develop the QSPR model by first data collection, analyzing the physico-chemical properties, dataset splitting, model training by various machine learning algorithms and evaluating final model on test set. Right panel shows the selected final QSPR model and the application to the central nervous system (CNS) physiologically based pharmacokinetic model (PBPK), the LeiCNS-PK-3.0 [6].

A systematic database of microdialysis-based K_p,uu,BBB_ values was created. For each compound, physico-chemical parameters were derived. The data was then split into train and test sets, with the train set being used to test several machine learning algorithms. The final model was evaluated and applied within the LeiCNS-PK3.0 model for selected compounds (Fig. 1).

### Data Set Collection of K_p,uu,BBB_

To acquire *in vivo* measured K_p,uu,BBB_ values, an extensive literature research was performed on studies utilizing the microdialysis approach in rat CNS. The microdialysis method was considered adequate if the microdialysis probe collected dialysate of the brain_ECF_ were corrected with the relative recovery, to convert the drug concentrations to unbound. K_p,uu,BBB_ values were either directly taken from the publications or calculated from reported unbound concentrations at steady state in brain_ECF_ (C_ECF,u,SS_) and in plasma (C_plasma,u,SS_) (Eq. 1). The K_p,uu,BBB_ values could also be derived, by calculating using the clearance in and out of the brain_ECF_ by the Cl_in_ over Cl_out_. (Eq. 2) or by the area under the curve (AUC) of unbound compounds, from timepoint 0 to infinity, in brain_ECF_ (AUC_ECF,u,0→∞_) and plasma (AUC_plasma,u,0→∞_) (Eq. 3). To enlarge the input dataset for building the QSPR model, microdialysis studies measuring the AUC to a specific timepoint instead of calculating to infinity were included. The collected K_p,uu,BBB_ values were log transformed for the use in QSPR model development.

1 $${K}_{p,uu,BBB}= \frac{{C}_{ECF,u,ss}}{{C}_{plasma,u,ss}}$$

2 $${K}_{p,uu,BBB}= \frac{{Cl}_{in}}{{Cl}_{out}}$$

3 $${K}_{p,uu,BBB}=\frac{{AUC}_{ECF,u,0\to \infty }}{{AUC}_{plasma,\text{u},0\to \infty }}$$

### Calculating of Physico-Chemical Properties

The dataset of physico-chemical properties of the drugs was generated using MOE (version 2024.0601) [17]. The 3D molecular structures of the compounds were obtained in MOE software by applying 3D coordinates as signed distance field (sdf) obtained from PubChem 2023 [18]. Only for DAMGO and digoxin the 2D coordinated were used since these compounds were unstable or contained to many atoms respectively to generate the coordinates. The compounds were cleaned using the function “wash”, with protonation set to dominant at pH 7.4. This step was done to clean the compound by disconnecting metal groups in simple salts and keeping only the large molecular fragments and strong acids were deprotonated and strong bases protonated. As last step, to ensure that each compound is in its stable (lowest energy) conformation and thereby enhancing the quality and reliability of the compound descriptors an “energy minimization” step was conducted. During this process the compounds were subjected to energy minimization by using the Merck Molecular Force Field 94 (MMFF94x) with a gradient of 0.01 RMS Kcal/mol/A2. The MMFF94x force field is chosen over other force fields since it has a higher accuracy for small molecular compounds for which the LeiCNS-PK3.0 is developed [19, 20]. Energy minimization using MMFF94x optimizes the 3D structure of the compound, ensuring that it adopts a stable, low-energy conformation. The minimized structure reflects better the actual shape and distribution of a compound in a biological system. Moreover, the minimized structure allows for a more accurate calculation of physicochemical descriptors. A total of 375 physico-chemical properties were calculated.

### QSPR Model Development

#### Data Splitting and Preprocessing

The data set containing the logK_p,uu,BBB_ values and the physico-chemical properties was split into 80% to the train set and 20% into the test set by a random split. For compounds with a high similarity to the test set (Tanimoto similarity > 0.7 based on RDKit (2024.3.5) [21]) topological fingerprints were excluded from the training set. The remaining compounds were used for model building. Feature selection was performed on the training set. First the descriptors with zero variance were removed. Followed by calculating, identifying and removing highly correlated descriptor with Pearson correlation with a threshold of 0.8. After removing the near zero and highly correlated descriptors, one final step of feature selection on the remaining descriptors was conducted with the Boruta feature selection algorithm (version 8.0.0, maxRuns 500) on the training set. The Boruta feature selection algorithm identifies relevant features by iteratively comparing the importance of actual features with randomized shadow features, retaining those with consistently higher importance than the shadows [22]. To evaluate the overlap of the chemical space of the train and test set a principal component analysis (PCA) on the chemical feature matrix was performed.

#### Machine Learning Algorithms

Machine learning models were implemented in R (version 4.4.0) using the packages caret (version 6.0.94), kernlab (version 0.9.32) and spls (version 2.2.3), based on different machine learning algorithms. The feature values used as input were standard scaled. Several QSPR algorithms were evaluated, including random forest (RF), support vector machines with linear kernel (SVM), K-nearest neighbors (KNN), sparse partial least squares (SPLS) and partial least squares (PLS). For every algorithm, tuning parameters were optimized by defining a tuning grid for the mtry for RF, costs (C) for SVM, k for KNN, eta and K for SPLS and lastly ncomp for PLS. The algorithms are further modelled using tenfold cross validation and 200 repeats.

### QSPR Model Evaluation

The predictive performance of the five different machine learning algorithms were compared, based on the average cross-validated predictive performance (q^2^), and the ML algorithm with the best predictive performance was selected. The q^2^ is calculated as the correlation between the observed and predicted values and squaring the value (R package caret). The selected best performing ML algorithm was further evaluated by estimating the root mean squared error (RMSE) and R^2^ on the train and test set and by checking the percentage within the 2- and 3-fold error range. Furthermore, the test set is also used to evaluate this model by checking the statistical characteristics advised by Golbraikh and Tropsha [23]. These characteristics were an R^2^ above 0.6, coefficients of determination below 0.1 and the slopes *k* of the regression line through the origin between 0.85 and 1.15. An applicability domain analysis was performed by calculating the Mahalanobis distance with a probability value of 0.95 of test data on the mean of the train data. To evaluate if the final model performance is better than chance, Y-randomization was performed. The target variable logK_p,uu,BBB_ was randomly permuted. The dataset preprocessing, model building and evaluation (only on the selected algorithm) was repeated with the permuted dataset for 30 iterations. On the outliers additional physico-chemical properties analysis, check on their distribution in the chemical space within the PCA analysis were performed. The predictive descriptors within the QSPR model were identified by estimating the importance of each descriptor individually and ranking them using the variable importance function in the R package caret and evaluated the relation with observed logK_p,uu,BBB_ values.

### Integration into the LeiCNS-PK3.0 Model

To demonstrate the application of QSPR models in the LeiCNS-PK3.0 CNS PBPK model, we generated predictions for CNS PK profiles for acetaminophen, methotrexate, paliperidone, phenytoin, raclopride and risperidone, as we had available in house measured microdialysis data and observed K_p,uu,BBB_ values for these compounds. Plasma PK, physico-chemical properties, and measured and predicted K_p,uu,BBB_ values of these drugs were used as input parameters. Simulations with LeiCNS-PK3.0 model were conducted with the observed K_p,uu,BBB_ values, followed by simulations with predicted values while keeping the plasma PK and physico-chemical parameters fixed. The predicted brain_ECF_ profiles from both sets of simulations were overlaid with observed data points to evaluate the predictions on the basis of visual predictive checks.

## Results

### Preprocessing Data

Of the 375 included descriptor, 122 descriptors remained after near zero variance and co-correlated descriptors removal. On the remaining descriptors the Boruta feature selection was applied and resulted in 10 confirmed descriptors and 4 tentative and the remaining rejected. These 14 descriptors including 2D and 3D are further used for model building. Short descriptions of the included descriptors may be found in Supplementary Table 1.

### Collected Data on K_p,uu,BBB_

**Fig. 2:**
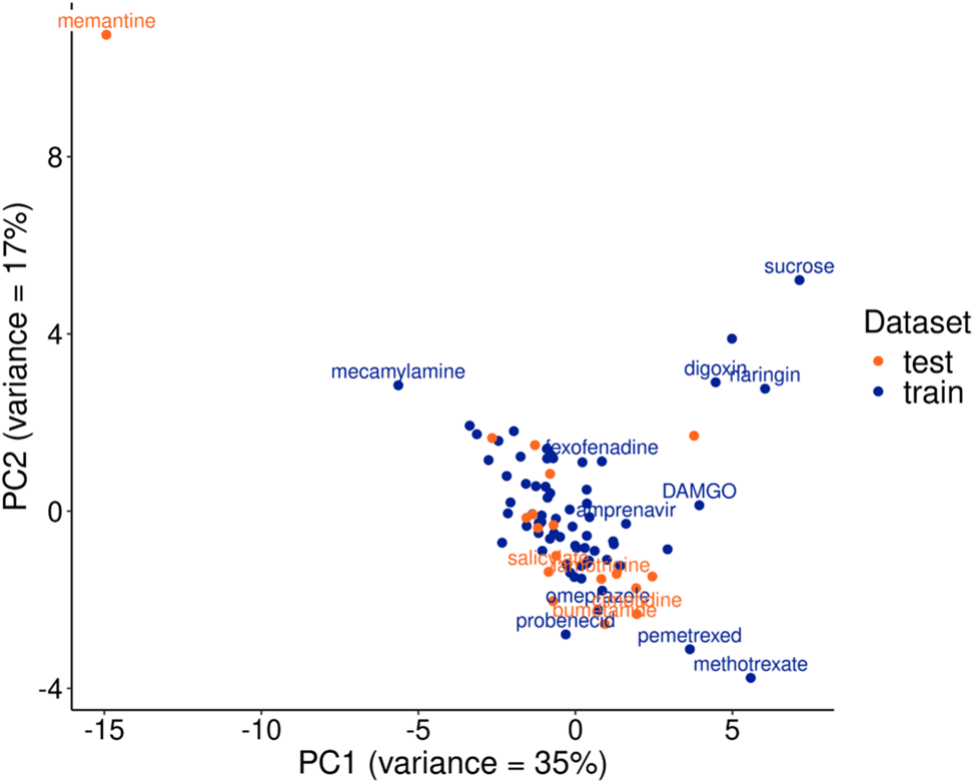
Principal Component Analysis (PCA) on the physico-chemical properties of the test and train set being used to train the machine learning algorithms. The results shows that the collected dataset with the partition coefficient, K_p,uu,BBB_, being divided in test and train set have overlapping chemical space. The annotated compounds were identified as outliers by using the Mahalanobis distance.

A total of 98 microdialysis-measured K_p,uu,BBB_ values were included for drugs with various physico-chemical properties (Supplementary Table 2–3). A Tanimoto similarity analysis, based on the topological fingerprints of the training set molecules, identified the 12 compounds in the train set as having high similarity (> 0.7) to compounds in the test set. To ensure independence of the test set, these 12 compounds were excluded from further model development. The chemical space of train and test set were compared by a PCA analysis within the first two principal components (PC) accounting for total 52% of the variance (PC1 = 35% and PC2 = 17*%).* Further analysis on the loadings of the descriptors within the first 2 PCs showed that H-bond donor capacity at −0.2 (vsurf_HB1) had the highest positive and Oprea leadlike test (opr_leadlike) the highest negative loading in PC1. Same analysis for PC2 showed that the descriptor for surface area of regions where the SMR values fall within a range of 0.11,0.26 (SMR_VSA1) had the highest positive load and number of nitrogen atoms (a_nN) the highest negative load. Appropriate overlap of the chemical space of train and test sets was observed (Fig. 2).

### QSPR Models

We trained five machine learning models on the train data set, using tenfold cross validation and 200 repeats for optimization of tuning parameters. The best performing model was selected based on the average cross-validated predictive performance, q^2^. Based on the predictive performance on train and test, the R^2^ and RMSE, the RF was selected as the best performing model with a q^2^ of 0.60 and RMSE of 0.48 (Table I). The worst predicting ML was the KNN with a q^2^ of 0.45 and RMSE of 0.58.

**Table I:** Overview of Predictive Performance of the Tested Machine Learning Algorithms on Train Set. The Results Shown are the Average Prediction Performances for the Train Data (*n* = 68) of the 10-fold Cross Validation and 200 Repeats with the Hyperparameter Optimized Tuning Parameters

Machine Learning Model	q^2^ (SD)	RMSE	Tuning parameter	Tuning range (steps)	R^2^
Random Forest (RF)	0.60 (0.26)	0.48	mtry = 2	1 – 14 (1)	0.61
Support Vector Machines (SVM)	0.60 (0.28)	0.15	C = 1	1 – 10 (1)	0.53
K-nearest neighbors (KNN)	0.45 (0.28)	0.58	k = 9	1 – 100 (1)	0.48
Sparse Partial Least Squares (SPLS)	0.56 (0.28)	0.56	eta = 0.95 K = 7	eta = 0.8 – 0.95 (0.01),K = 1 – 10	0.27
Partial Least Squares (PLS)	0.60 (0.28)	0.49	ncomp = 1	1 – 10 (1)	0.26

q^2^ = average cross validated prediction error, R^2^ = prediction error, RMSE = root mean square error. SD = standard deviation

### The Random Forest Model Performance Testing

**Fig. 3:**
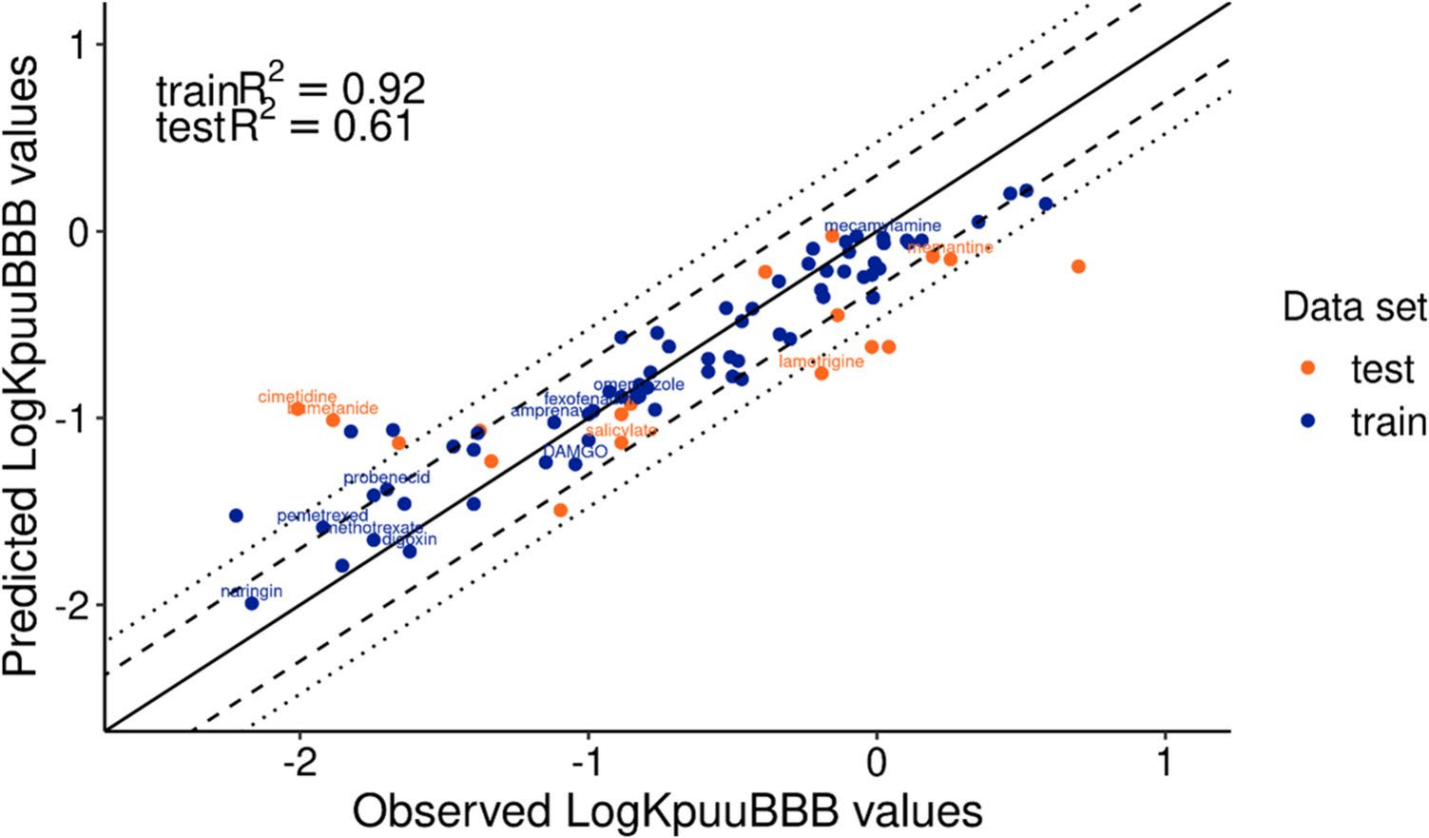
Observed *versus* predicted logK_p,uu,BBB_ values with Random Forest model for train and test data. The prediction error (R^2^) of both datasets is given in the left top corner. The solid line is the regression line, the dashed line is 2-fold error indication and dotted line is 3-fold error indication. The compounds with labels are the outliers identified with the Mahalanobis distance.

The RF model performance was further evaluated by evaluating the predictive performance on train and test set. The predictive performance, R^2^ of test was 0.61 with an RMSE of 0.52 (Table II). For the train set the R^2^ was 0.92 and the RMSE of 0.24. The Y-randomization validation (25 iterations, five iterations were skipped, due to 0 descriptors being selected) showed an R^2^ on the test set of 0.11 (SD 0.14) and on train set of 0.85 (SD 0.09). The predicted values were calculated and the percentage within the twofold and threefold errors were determined and of the test set 33.3% was within twofold and 61.1% within threefold errors. The train set predictions was for 79.4% within the twofold and 94.1% within the threefold errors. The observed *versus* predicted logK_p,uu,BBB_ values (Fig. 3) of the final model demonstrate that mainly values around predicted logK_p,uu,BBB_ of −1 were outside the threefold change error range. The Mahalanobis distance applicability domain analysis identified 16 outliers in the whole dataset.

**Table II:** Random Forest Train and Test Data Performances and R^2^ on Y-randomization with 30 Iterations

Random Forest	R^2^	RMSE	Within twofold error (%)	Within threefold error (%)	Y-randomized R^2^ mean (SD)*	Y-randomized q^2^ mean (SD)*
Train (*n* = 68)	0.92	0.24	79.4	94.1	0.86 (0.07)	0.27 (0.06)
Test (*n* = 18)	0.61	0.52	33.3	61.1	0.08 (0.08)	

*five iterations were skipped, due to 0 descriptors being selected. SD = standard deviation

**Fig. 4:**
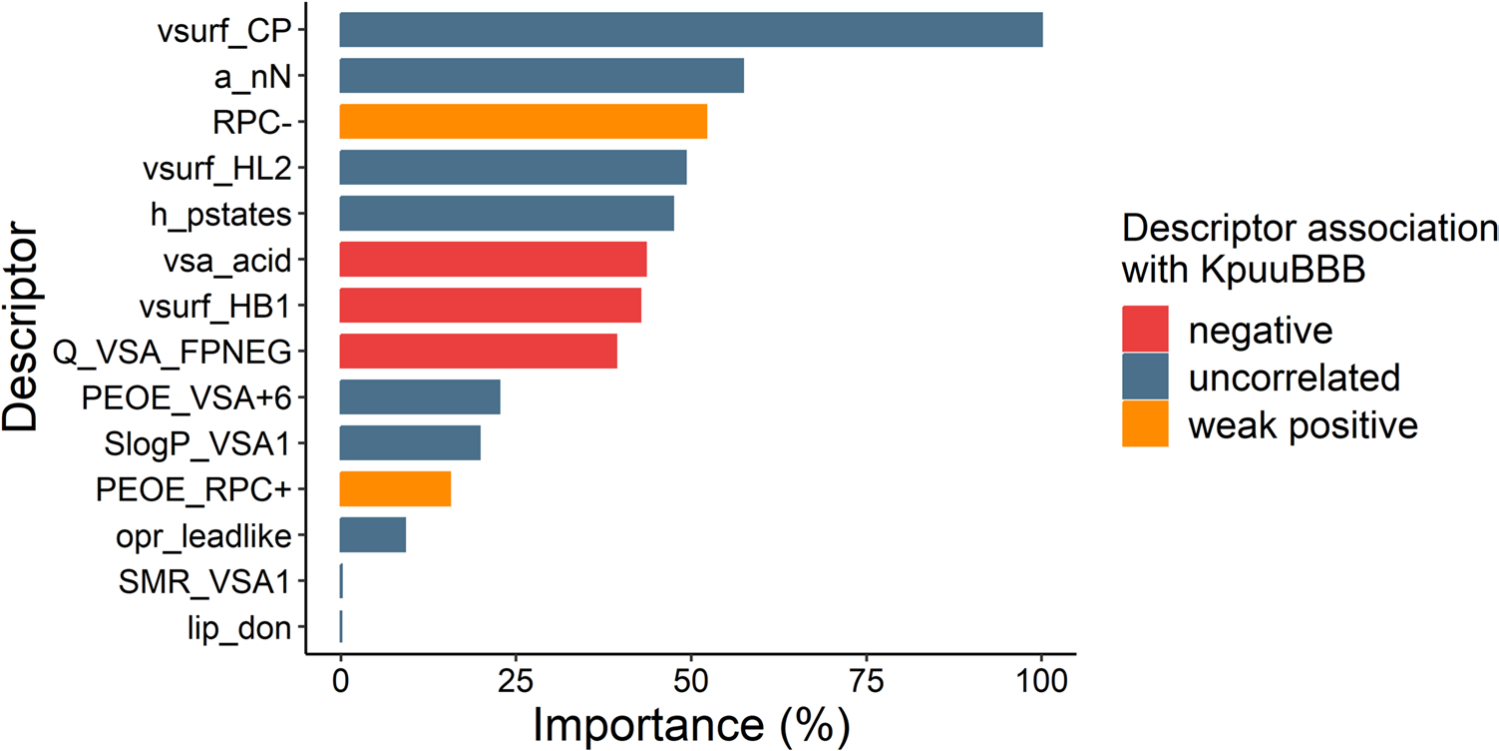
Ranked chemical descriptors (top 5) for the final Random Forest model in decreasing order. The most important descriptors of the random forest model for prediction of the partition coefficient, K_p,uu,BBB_, ordered from highest to lowest importance value (%). Bars are colored according to their association with K_p,uu,BBB_: blue bars indicate a positive association (higher descriptor value correspond to higher K_p,uu,BBB_ value) and red bars indicate a negative association (higher descriptor value correspond to lower K_p,uu,BBB_ value). vsurf_CP = VolSurf Critical packing parameter, a_nN = number of nitrogen atoms, RPC- = relative negative partial charge, vsurf_HL2 = Second hydrophilic-lipophilic balance descriptor, h_pstates = entropic state count at pH 7, vsa_acid = approximation of Van der Waals surface area of acidic regions in the molecule,vsurf_HB1 = hydrogen bond donor capacity, Q_VSA_FPNEG = fractional negative polar van der Waals surface area, PEOE_VSA + 6 = sum of van der Waals surface areas of atoms within a partial charge, SlogP_VSA1 = surface area descriptor associated with Log of the octanol/water partition coefficient, PEOE_RPC +  = relative positive partial charge, opr_leadlike = the Oprea Lead-like test, SMR_VSA1 = sum of van der Waals surface areas for atoms within a range for molar refractivity, lip_don = the count of hydrogen bond donors.

The top three most important chemical characteristics (Fig. 4) in the final RF models included the VolSurf Critical packing parameter (vsurf_CP), number of nitrogen atoms (a_nN) and relative negative partial charge (RPC-). Individual analysis of the descriptors and logK_p,uu,BBB_ values showed for RPC- an overall weak positive association and for the other two no correlation was observed.

### Integration into the LeiCNS-PK3.0 Model

**Fig. 5:**
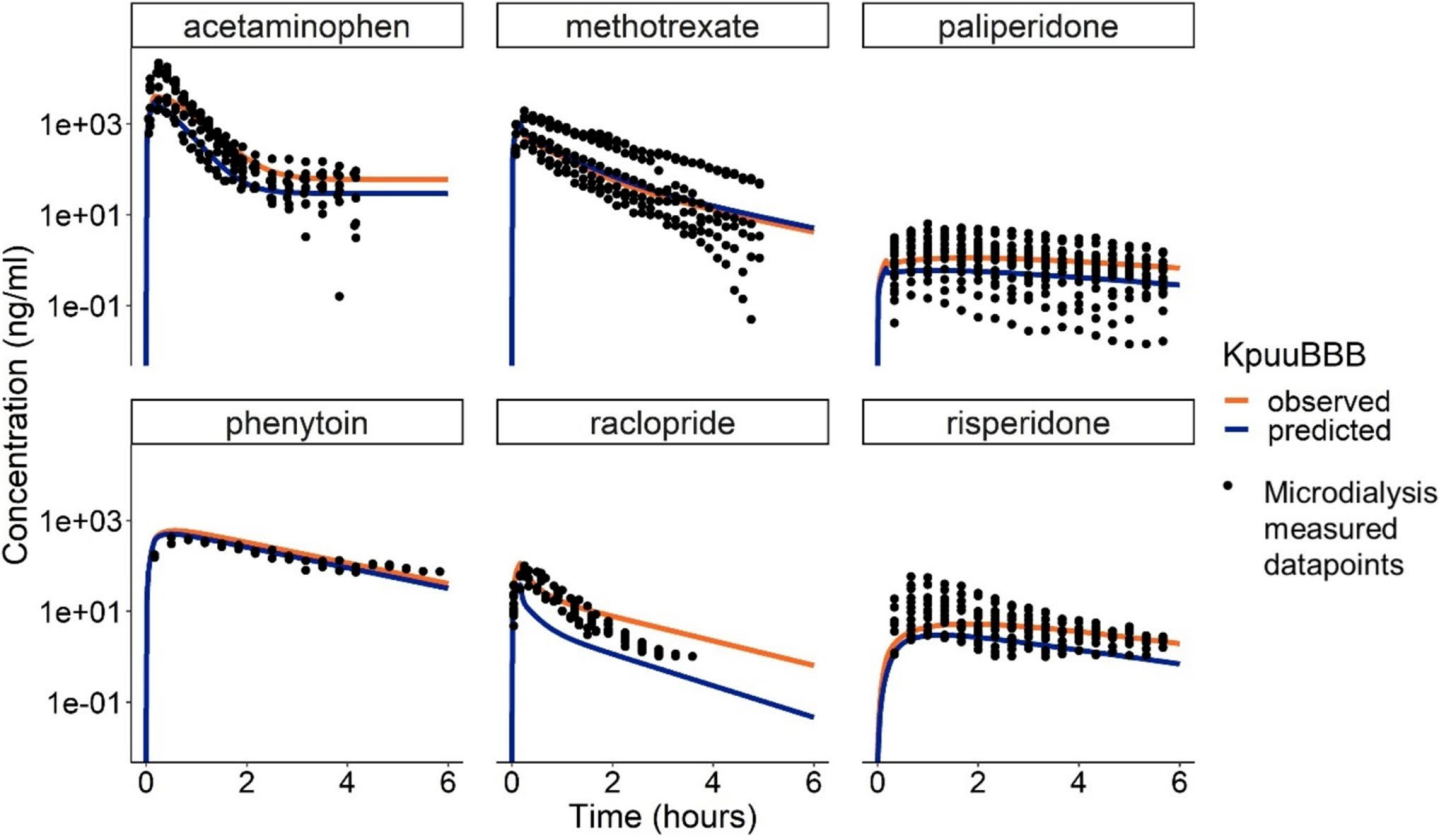
Predicted concentration–time profiles of six drugs at the rat brain extracellular fluid (brain_ECF_). Simulations were performed with the observed and predicted K_p,uu,BBB_ value for acetaminophen, methotrexate, paliperidone, phenytoin, raclopride and risperidone. Acetaminophen and raclopride were in the test set and the other four in the train set. The simulations were completed using the central nervous system physiologically based pharmacokinetic, LeiCNS-PK3.0, model. The drug profiles were compared to inhouse microdialysis measured rat studies (black dots).

To demonstrate the integration of the QSPR model into the LeiCNS-PK3.0 model, acetaminophen, methotrexate, paliperidone, phenytoin, raclopride and risperidone were used for predicting the distribution in the rat brain_ECF_ (Fig. 5). Of these drugs acetaminophen and raclopride were in the test set while the other four were in the train set. The simulations were conducted with observed and QSPR model predicted K_p,uu,BBB_ values and the LeiCNS-PK3.0 predicted concentration profiles were evaluated against in house measured microdialysis observations. LeiCNS-PK3.0 simulations using the QSPR predicted K_p,uu,BBB_ that for most of the drugs, except raclopride, the predictions were overlapping for with the rat microdialysis observations.

## Discussion

In this study we successfully developed a QSPR model using a random forest regression model. This model was able to predict for more than 61% of K_p,uu,BBB_ values within a threefold error, using a unique large-scale dataset based on microdialysis measurements. In addition, we demonstrated how the developed QSPR model could be integrated into a CNS PBPK modeling workflow for prediction of CNS drug disposition.

The developed QSPR model showed a predictive performance with an R^2^ of 0.61 for the test set. This performance is in line with previously developed QSPR models (R^2^ = 0.53 – 0.89) [9–16]. The predictive performance of our model on the cross-validated training was associated with a q^2^ of 0.60 and an R^2^ value 0.61. Y-randomization analysis showed a low R^2^ of 0.11 indicating limited overfitting. Furthermore, performance of our model was superior in comparison to a previously published QSPR model developed with a smaller dataset (n = 53) and lower predictive performance (test R^2^ = 0.35) [16]. The Mahalanobis distance identified 5 outliers within the test set and visually comparing these outliers in the PCA with the train set outliers showed overlap. Moreover, the identified outliers within the test set appear to have a predicted logK_p,uu,BBB_ around −1, except for memantine. The other compounds in the test set were generally close to the regression line. These finding suggest that the QSPR model developed could be applicable to the compounds chemically close to the ones within the applicability domain and makes this QSPR model suitable for further applications as part of PBPK workflows.

In this study, we utilized K_p,uu,BBB_ values obtained exclusively through microdialysis for constructing the QSPR model. In contrast, most existing QSPR models use K_p,uu,brain_ values that use fraction unbound in brain by brain homogenate assays, and fraction unbound in plasma protein by *in vitro* equilibrium dialysis methods. These approaches do not take into account the intact physiological context of the processes that govern CNS drug distribution. Microdialysis based K_p,uu,BBB_ values are most relevant, as these are obtained *in vivo*, in the full physiological context. Therefore we consider the microdialysis derived K_p,uu,BBB_ values as the best reflecting the real situation and used selectively these in our QSPR model development. Only one previous study has built a QSPR model based on microdialysis data, but it tested on a relative small number of 53 compounds [16]. Our study, however, developed a QSPR model by testing a larger number of compounds, resulting in a broader chemical space and higher predictive performance without the need to incorporate additional information on BBB transporters. The enhanced predictive performance suggests the usability of the developed QSPR model in clinically relevant predictions.

A previous study on comparing the predictive performance in building QSPR models using 2D and 3D descriptors suggested that using both will increase the predictive accuracy and in predicting the clinical success [24, 25]. The inclusion of large amount of 2D or 3D descriptors gave us the possibility to train the machine learning algorithms on various properties of the compounds and learn more about the potential relevant chemical features. In this study a number of important descriptors were identified which can impact the K_p,uu,BBB_ of a drug and thereby transport across the BBB. Comparing the important descriptors obtained with the QSPR model in this study and others showed some overlapping descriptors. In this study descriptors as the ionization potential, charge, energy, number of nitrogen atoms, hydrogen bonding and hydrophilicity of the compounds were identified as important. This was partially in line with previous studies who identified hydrogen bonding, molecular topology, polar surface area and molecular volume as important descriptors for K_p,uu,BBB_ prediction. Overlapping parameters such as the polar surface area and hydrogen bonding have been suggested to correlate with P-gp interaction, and the charge, due to the negative charge of the BBB, may to rely on other active transport mechanisms [26–28]. Another descriptor from our study, the number of nitrogen atoms, have been studied before and the results of that study showed that various nitrogenous substructures can facilitate BBB penetration on different levels [29]. The ionization potential identified so far only in our study is a measure for the energy required to remove an electron from the molecule to become charged. The higher the ionization potential, the more energy is needed for the molecule to become charged, less polar and more lipophilic, which could be beneficial for passive transport and avoiding active transport as efflux transport. Our study confirmed the importance of the already identified descriptors and in addition showed the importance of other descriptors.

The currently developed QSPR model could be useful aid for design and development of early stages in CNS active drug with intended or not intended CNS effects, and CNS toxicity. Its application in predicting the K_p,uu,BBB_ value for the LeiCNS-PK3.0 model accelerates and broadens the model’s use, reducing the need for animal testing and predicting PK profiles in various physiological compartments, including drug target sites. We show for the six drugs included in our simulations, the predicted brain_ECF_ PK profiles are mostly in line with the microdialysis measured concentrations. The combination of QSPR and CNS PBPK modeling further opens up the possibility to investigate what physico-chemical properties would results in good PK profiles for optimal target engagement, as well as predictions of drug distribution in other species, healthy and disease conditions or any other physiological changed conditions [30, 31].

We expect further refinements and improvements of QSPR model predictions may be achieved by adding drug-transporter interaction information. Previous studies adding transport information as P-glycoprotein efflux ratios in a mice QSPR model and adding influx and efflux transporter information in a SPLS algorithm model showed improvement in the model performance [8, 14, 16]. In addition, some of the compounds can undergo metabolism at the BBB for these additional interaction with metabolic enzymes can be added. With additional interactions the current QSPR model could be improved and better predictions can be achieved.

In conclusion, in this study we developed an QSPR model with acceptable predictive performance in prediction of K_p,uu,BBB_ values. Beside using this model for prediction of the extent of drug distribution, the predicted K_p,uu,BBB_ value can be used as input into the LeiCNS-PK3.0 model to predict the unbound CNS drug distribution in various physiological conditions.

## Supplementary Information

Below is the link to the electronic supplementary material.

Supplementary file1 (DOCX 218 KB)

## Data Availability

The data supporting the findings of this study are available within the article and its supplementary materials. The code is not publicly available due to intellectual property restrictions.
