# Supplementary material for: Prediction of the Extent of Blood–Brain Barrier Transport Using Machine Learning and Integration into the LeiCNS-PK3.0 Model
Source: Pharm Res. 2025 Feb 10;42(2):281–9. doi: 10.1007/s11095-025-03828-0 (PMC11880073; doi:10.1007/s11095-025-03828-0)
Supplement: Supplementary file 1 — Supplementary file1 (DOCX 218 KB) [file 11095_2025_3828_MOESM1_ESM.docx]

**Prediction of the extent of blood-brain barrier transport using machine learning and integration into the LeiCNS-PK3.0 model.**

Berfin Gülave, Helle W. van den Maagdenberg*, Luke van Boven*, Gerard J.P. van Westen, Elizabeth C.M. de Lange, J.G. Coen van Hasselt

*These authors contributed equally to the work


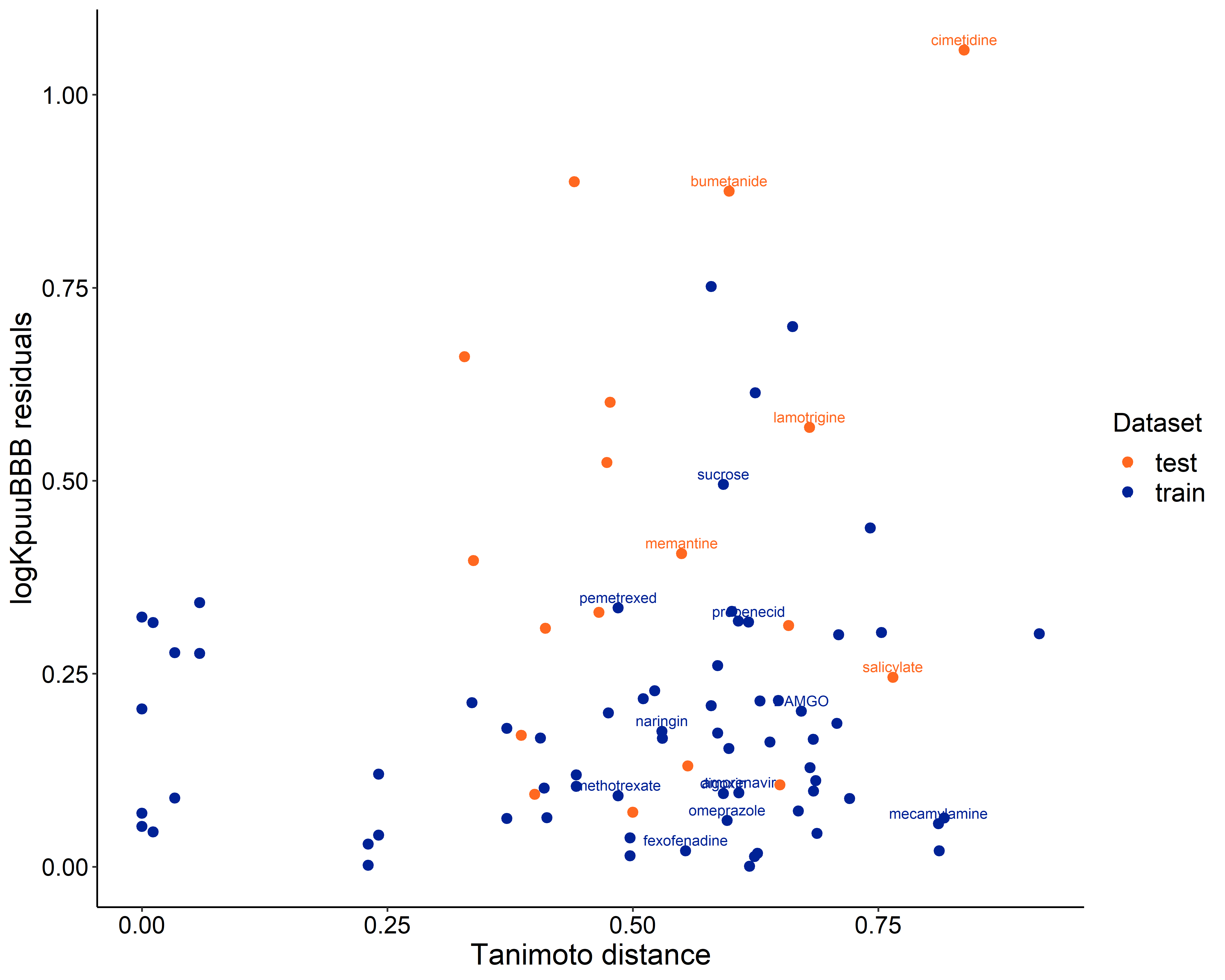


**Supplementary figure 1 LogK_p,uu,BBB_ residuals versus the Tanimoto distance plot.** The residuals represent the absolute difference between the predicted and observed logK_p,uu,BBB_ values. The Tanimoto difference is calculated as 1 – Tanimoto similarity which is based RDKit topological fingerprints as similarity of test set on training set. The labeled compounds are the with the Mahalanobis distance identified outliers.

**Supplementary Table 1. The list of descriptors used for the various machine learning algorithms obtained from Molecular Operating Environment (MOE) with their short description and class together with the Boruta feature selection (BFS) decision.**

| **Physicochemical properties** | **Class** | **Description** | **BFS** |
| --- | --- | --- | --- |
| a_nN | 2D | Number of nitrogen atoms, characterizing molecular composition | C |
| h_pstates | 2D | Entropic state count at pH 7, representing the protonation state of the compound | C |
| lip_don | 2D | The count of hydrogen bond donors based on Lipinski's Rule of Five | T |
| opr_leadlike | 2D | Binary descriptor indicating whether it follows (less than 2 violations) the Oprea Lead-like Test | T |
| PEOE_RPC+ | 2D | Relative positive partial charge derived from PEOE method (charge is transferred between bonded atoms until equilibrium) | C |
| PEOE_VSA+6 | 2D | Sum of van der Waals surface areas of atoms with a partial charge between 0.30 and 0.35, providing further detail on surface charge distribution. | T |
| Q_VSA_FPNEG | 2D | Fractional negative polar van der Waals surface area, showing the extent of the surface that is negatively charged. | C |
| RPC- | 2D | Relative negative partial charge, indicative of electron-rich regions. | C |
| SlogP_VSA1 | 2D | Surface area descriptor associated with Log of the octanol/water partition coefficient within a range of -0.4,-0.2, indication contribution of specific molecular regions to hydrophobicity. | C |
| SMR_VSA1 | 2D | Sum of van der Waals surface areas for atoms with a molar refractivity in a range of 0.11-0.26, giving insight into molecular polarizability. | T |
| vsa_acid | 2D | Approximation of Van der Waals surface area of acidic regions in the molecule, indicating areas capable of donating protons under physiological conditions. | C |
| vsurf_CP | 3D | VolSurf Critical packing parameter, indicating molecular compactness and packing density. | C |
| vsurf_HB1 | 3D | Hydrogen bond donor capacity derived from the VolSurf methodology, quantifying the hydrophobicity of a molecule. | C |
| vsurf_HL2 | 3D | Second hydrophilic-lipophilic balance descriptor, highlighting the balance between hydrophilic and hydrophobic regions. | C |
| *BFS = decision on the descriptor by Boruta feature selection* (1)  *C = confirmed*  *T = tentative* | | | |

**Supplementary table 2. List of included drugs with the observed and predicted K_p,uu,BBB_ values within the training set.**

| **Drug** | **K_p,uu,BBB_** | | **Ref** |
| --- | --- | --- | --- |
|  | Predicted | Observed |  |
| acyclovir* | 0.17 | 0.316 | (2) |
| alovudine | 0.18 | 0.164 | (3) |
| alprenolol | 0.61 | 0.77 | (4) |
| ambroxol | 0.39 | 0.3 | (5) |
| amprenavir | 0.09 | 0.076 | (6) |
| antipyrine | 0.86 | 1.057 | (7) |
| apomorphine-R^#^ | - | 10.75 | (8) |
| atenolol | 0.15 | 0.16 | (4) |
| baicalein* | 0.03 | 0.023 | (9) |
| bupivacaine^#^ | - | 0.582 | (10) |
| bupropion | 1.65 | 3.3 | (11) |
| camptothecin | 0.18 | 0.26 | (12) |
| carbamazepine | 0.63 | 1.02 | (13) |
| cefazolin^#^ | - | 0.06 | (14) |
| ceftazidime^#^ | - | 0.026 | (15) |
| ceftriaxone^#^ | - | 0.053 | (15) |
| citalopram | 0.89 | 1.43 | (16) |
| clozapine | 0.09 | 0.021 | (17) |
| cocaine | 0.38 | 0.37 | (18) |
| codeine^#^ | - | 1 | (13) |
| colchicine* | 0.07 | 0.04 | (19) |
| DAMGO | 0.06 | 0.09 | (20) |
| diazepam | 0.68 | 0.982 | (21) |
| digoxin | 0.02 | 0.024 | (7) |
| diltiazem | 0.57 | 0.9 | (7) |
| diphenhydramine | 1.40 | 3.85 | (22) |
| duloxetine | 0.81 | 0.6 | (23) |
| escitalopram | 0.88 | 0.78 | (24) |
| fexofenadine | 0.11 | 0.104 | (7) |
| fluorescein | 0.04 | 0.018 | (25) |
| fluoxetine^#^ | - | 1.25 | (16) |
| ganciclovir | 0.06 | 0.18 | (7) |
| genistein* | 0.03 | 0.04 | (26) |
| granisetron | 0.27 | 0.13 | (27) |
| imipramine | 0.08 | 0.015 | (28) |
| indomethacin | 0.08 | 0.1 | (29) |
| levetiracetam | 0.21 | 0.31 | (30) |
| levofloxacin | 0.16 | 0.34 | (31) |
| lidocaine | 0.54 | 0.457 | (10) |
| mannitol | 0.02 | 0.014 | (32) |
| mecamylamine | 0.92 | 1.05 | (16) |
| 6-mercaptopurine* | 0.08 | 0.0413 | (33) |
| methotrexate | 0.02 | 0.018 | (29) |
| metoclopramide | 0.61 | 0.669 | (34) |
| metoprolol | 0.49 | 0.64 | (4) |
| midazolam | 0.44 | 0.652 | (34) |
| mitragynine | 0.67 | 0.579 | (35) |
| morphine^#^ | - | 0.38 | (29) |
| M6G^#^ | - | 0.29 | (36) |
| naltrexone^#^ | - | 0.407 | (37) |
| naringin | 0.01 | 0.0068 | (38) |
| norfloxacin | 0.07 | 0.034 | (39) |
| nortriptyline | 0.94 | 0.85 | (16) |
| ofloxacin | 0.14 | 0.118 | (39) |
| omeprazole | 0.13 | 0.15 | (40) |
| ondansetron | 0.24 | 0.19 | (4) |
| oxycodone^#^ | - | 2.9 | (41) |
| paliperidone | 0.26 | 0.5 | (29) |
| paroxetine | 0.87 | 1.29 | (16) |
| pefloxacin | 0.13 | 0.147 | (39) |
| pemetrexed | 0.03 | 0.012 | (7) |
| phenytoin | 0.21 | 0.26 | (29) |
| pindolol | 0.33 | 0.34 | (4) |
| pregabalin | 0.10 | 0.1 | (42) |
| probenecid | 0.04 | 0.02 | (43) |
| quinidine | 0.29 | 0.173 | (37) |
| remoxipride | 0.77 | 0.8 | (44) |
| risperidone | 0.44 | 0.97 | (29) |
| sertraline | 0.89 | 1.27 | (16) |
| stavudine | 0.20 | 0.33 | (45) |
| sucrose | 0.01 | 0.0027 | (46) |
| sumatriptan | 0.13 | 0.13 | (7) |
| temozolomide | 0.28 | 0.46 | (47) |
| tetramethylpyrazine* | 1.12 | 2.25 | (48) |
| theophylline^#^ | - | 0.99 | (7) |
| tiagabine | 0.03 | 0.006 | (6) |
| tolcapone | 0.11 | 0.17 | (4) |
| tramadol | 1.59 | 2.9 | (22) |
| varenicline | 0.58 | 0.96 | (16) |
| zidovudine | 0.15 | 0.15 | (45) |
| ** The K_p,uu,BBB_ values are not determined at steady state neither the area under the curve values are extrapolated to infinity.*  *# These compounds are excluded since they have a Tanimoto similarity higher of 0.7* | | | |

**Table 3 List of included drugs with the observed and predicted K_p,uu,BBB_ values within the test set.**

| **Drug** | **K_p,uu,BBB_** | | **Ref** |
| --- | --- | --- | --- |
|  | Predicted | Observed |  |
| acetaminophen | 0.36 | 0.73 | (11) |
| apomorphine-S | 0.65 | 5 | (8) |
| atomoxetine | 0.95 | 0.7 | (49) |
| bumetanide | 0.10 | 0.013 | (50) |
| caffeine | 0.24 | 0.96 | (32) |
| cefadroxil | 0.07 | 0.022 | (51) |
| cefuroxime* | 0.09 | 0.042 | (52) |
| cimetidine | 0.11 | 0.104 | (7) |
| entacapone | 0.12 | 0.14 | (4) |
| gabapentin | 0.10 | 0.13 | (13) |
| ketoprofen | 0.06 | 0.12 | (53) |
| lamotrigine | 0.17 | 0.643 | (34) |
| levobupivacaine | 0.61 | 0.41 | (10) |
| memantine | 0.71 | 1.8 | (22) |
| M3G | 0.03 | 0.08 | (54) |
| propranolol | 0.73 | 1.562 | (7) |
| raclopride | 0.24 | 1.1 | (44) |
| salicylate | 0.07 | 0.13 | (43) |

**References**

1. Kursa MB, Rudnicki WR. Feature selection with the boruta package. J Stat Softw. 2010;36(11):1–13.

2. Shan Y, Cen Y, Zhang Y, Tan R, Zhao J, Nie Z, et al. Acyclovir Brain Disposition: Interactions with P-gp, Bcrp, Mrp2, and Oat3 at the Blood–Brain Barrier. Eur J Drug Metab Pharmacokinet [Internet]. 2022;47(2):279–89. Available from: https://doi.org/10.1007/s13318-021-00733-w

3. Stahle L BN. Transport of alovudine (3′-fluorothymidine) into the brain and the cerebrospinal fluid of the rat, studied by microdialysis. Life Sci. 2000;66(19):1805–16.

4. Hakkarainen JJ, Jalkanen AJ, Kääriäinen TM, Keski-Rahkonen P, Venäläinen T, Hokkanen J, et al. Comparison of in vitro cell models in predicting in vivo brain entry of drugs. Int J Pharm. 2010 Dec;402(1–2):27–36.

5. Yang CN, Peng WY, Lin LC, Tsai TH. Protein unbound pharmacokinetics of ambroxol in the blood and brains of rats and the interaction of ambroxol with Polygala tenuifolia by multiple microdialysis. J Ethnopharmacol. 2021 Apr;269.

6. Edwards JE, Brouwer KR, McNamara PJ. GF120918, a P-glycoprotein modulator, increases the concentration of unbound amprenavir in the central nervous system in rats. Antimicrob Agents Chemother. 2002;46(7):2284–6.

7. Chen C, Zhou H, Guan C, Zhang H, Li Y, Jiang X, et al. Applicability of free drug hypothesis to drugs with good membrane permeability that are not efflux transporter substrates: A microdialysis study in rats. Pharmacol Res Perspect. 2020 Apr;8(2).

8. Sam E, Sarre S, Michotte Y, Verbeke N. Distribution of apomorphine enantiomers in plasma, brain tissue and striatal extracellular fluid. Eur J Pharmacol. 1997;329(1):9–15.

9. Tsai TH, Liu SC, Tsai PL, Ho LK, Shum AYC, Chen CF. The effects of the cyclosporin A , a P-glycoprotein inhibitor , on the pharmacokinetics of baicalein in the rat : a microdialysis study. Br J Pharmacol. 2002;137:1314–20.

10. Ikeda Y, Oda Y, Nakamura T, Takahashi R, Miyake W, Hase I, et al. Pharmacokinetics of lidocaine, bupivacaine, and levobupivacaine in plasma and brain in awake rats. Anesthesiology. 2010;112(6):1396–403.

11. Summerfield SG, Zhang Y, Liu H. Examining the uptake of central nervous system drugs and candidates across the blood-brain barrier. J Pharmacol Exp Ther. 2016;358(2):294–305.

12. Tsai TH, Lee CH, Yeh PH. Effect of P-glycoprotein modulators on the pharmacokinetics of camptothecin using microdialysis. Br J Pharmacol. 2001;134:1245–52.

13. Hammarlund-Udenaes M. The use of microdialysis in CNS drug delivery studies: Pharmacokinetic perspectives and results with analgesics and antiepileptics. Adv Drug Deliv Rev. 2000;45(2–3):283–94.

14. Tsai TH, Chen Y. Simultaneous determination of cefazolin in rat blood and brain by microdialysis and microbore liquid chromatography. | Semantic Scholar. Biomedical Chromatography. 2000. p. 274–8.

15. Granero L, Santiago M, Cano J, Machado A, Peris JE. Analysis of ceftriaxone and ceftazidime distribution in cerebrospinal fluid of and cerebral extracellular space in awake rats by in vivo microdialysis. Antimicrob Agents Chemother. 1995;39(12):2728–31.

16. Weber ML, Hofland CM, Shaffer CL, Flik G, Cremers T, Hurst RS, et al. Therapeutic doses of antidepressants are projected not to inhibit human α4β2 nicotinic acetylcholine receptors. Neuropharmacology. 2013;72:88–95.

17. Hou ML, Lin CH, Lin LC, Tsai TH. The Drug-Drug Effects of Rhein on the Pharmacokinetics and Pharmacodynamics of Clozapine in Rat Brain Extracellular Fluid by In Vivo Microdialysis. J Pharmacol Exp Ther. 2015 Oct;355(1):125–34.

18. Chen Y, Chang C, Wang S, Tsai T. Measurement of unbound cocaine in blood , brain and bile of anesthetized rats using microdialysis coupled with liquid chromatography and verified by tandem mass spectrometry. Biomed Chromatogr. 2005;408:402–8.

19. Desrayaud S, Guntz P, Scherrmann JM LM. Effect of the P-glycoprotein inhibitor, SDZ PSC 833, on the blood and brain pharmacokinetics of colchicine. Life Sci. 1997;61(2):153–63.

20. Lindqvist A, Rip J, Gaillard PJ, Hammarlund-udenaes M. Enhanced Brain Delivery of the Opioid Peptide DAMGO in Glutathione PEGylated Liposomes: A Microdialysis Study. 2013;

21. Dubey RK, McAllister CB, Inoue M, Wilkinson GR. Plasma binding and transport of diazepam across the blood-brain barrier. No evidence for in vivo enhanced dissociation. J Clin Invest. 1989;84(4):1155–9.

22. Kitamura A, Okura T, Higuchi K, Deguchi Y. Cocktail-Dosing Microdialysis Study to Simultaneously Assess Delivery of Multiple Organic-Cationic Drugs to the Brain. J Pharm Sci [Internet]. 2016;105(2):935–40. Available from: http://dx.doi.org/10.1002/jps.24691

23. Kielbasa W, Stratford RE. Exploratory translational modeling approach in drug development to predict human brain pharmacokinetics and pharmacologically relevant clinical doses. Drug Metab Dispos. 2012;40(5):877–83.

24. Bundgaard C, Jørgensen M, Larsen F. Pharmacokinetic modelling of blood–brain barrier transport of escitalopram in rats. Biopharm Drug Dispos. 2007 Oct;28(7):349–60.

25. Sun H, Miller DW, Elmquist WF. Effect of Probenecid on Fluorescein Transport in the Central Nervous System Using In Vitro and In Vivo Models. Pharm Res. 2001;18(11):1542–9.

26. Tsai T. Concurrent measurement of unbound genistein in the blood , brain and bile of anesthetized rats using microdialysis and its pharmacokinetic application. J Chromatogr A. 2005;1073:317–22.

27. Huang CT, Chen CF, Tsai TH. Pharmacokinetics of granisetron in rat blood and brain by microdialysis. Life Sci. 1999 Apr;64(21):1921–31.

28. O’Brien FE, Clarke G, Fitzgerald P, Dinan TG, Griffin BT, Cryan JF. Inhibition of P-glycoprotein enhances transport of imipramine across the blood-brain barrier: microdialysis studies in conscious freely moving rats. Br J Pharmacol. 2012 Jun;166(4):1333–43.

29. Saleh MAA, Loo CF, Elassaiss-Schaap J, De Lange ECM. Lumbar cerebrospinal fluid-to-brain extracellular fluid surrogacy is context-specific: insights from LeiCNS-PK3.0 simulations. J Pharmacokinet Pharmacodyn [Internet]. 2021;48(5):725–41. Available from: https://doi.org/10.1007/s10928-021-09768-7

30. Saleh MAA, de Lange ECM. Impact of cns diseases on drug delivery to brain extracellular and intracellular target sites in human: A “what-if” simulation study. Pharmaceutics. 2021;13(1):1–17.

31. Cen Y, Shan Y, Zhao J, Xu X, Nie Z, Zhang J. Multiple drug transporters contribute to the brain transfer of levofloxacin. CNS Neurosci Ther. 2023 Jan;29(1):445–57.

32. Hansen DK, Scott DO, Otis KW, Lunte SM. Comparison of in vitro BBMEC permeability and in vivo CNS uptake by microdialysis sampling. J Pharm Biomed Anal. 2002 Mar;27(6):945–58.

33. Deguchi Y, Y Yokoyama, T Sakamoto, H Hayashi, T Naito, S Yamada RK. Brain distribution of 6-mercaptopurine is regulated by the efflux transport system in the blood-brain barrier. Life Sci. 2000;66(7):649–62.

34. Nagaya Y, Nozaki Y, Takenaka O, Watari R, Kusano K, Yoshimura T, et al. Investigation of utility of cerebrospinal fluid drug concentration as a surrogate for interstitial fluid concentration using microdialysis coupled with cisternal cerebrospinal fluid sampling in wild-type and Mdr1a(-/-) rats. Drug Metab Pharmacokinet [Internet]. 2016;31(1):57–66. Available from: http://dx.doi.org/10.1016/j.dmpk.2015.10.003

35. Kong WM, Mohamed Z, Alshawsh MA, Chik Z. Evaluation of pharmacokinetics and blood-brain barrier permeability of mitragynine using in vivo microdialysis technique. J Pharm Biomed Anal [Internet]. 2017;143:43–7. Available from: http://dx.doi.org/10.1016/j.jpba.2017.05.020

36. Tunblad K, Hammarlund-Udenaes M, Jonsson EN. Influence of probenecid on the delivery of morphine-6-glucuronide to the brain. Eur J Pharm Sci. 2005;24(1):49–57.

37. Durk MR, Deshmukh G, Valle N, Ding X, Liederer BM, Liu X. Use of subcutaneous and intraperitoneal administration methods to facilitate cassette dosing in microdialysis studies in rats. Drug Metab Dispos. 2018;46(7):964–9.

38. Tsai TH. Determination of Naringin in Rat Blood , Brain , Liver , and Bile Using Microdialysis and Its Interaction with Cyclosporin A , a P-Glycoprotein Modulator. J Agric Food Chemsitry. 2002;50(23):6669–74.

39. Ooie T, Terasaki T, Suzuki H SY. Quantitative brain microdialysis study on the mechanism of quinolones distribution in the central nervous system. Drug Metab Dispos. 1997;25(7):784–9.

40. Cheng FC, Ho YF, Hung LC, Chen CF, Tsai TH. Determination and pharmacokinetic profile of omeprazole in rat blood, brain and bile by microdialysis and high-performance liquid chromatography. J Chromatogr A. 2002 Mar;949(1–2):35–42.

41. Gustafsson S, Eriksson J, Syvänen S, Eriksson O, Hammarlund-Udenaes M, Antoni G. Combined PET and microdialysis for in vivo estimation of drug blood-brain barrier transport and brain unbound concentrations. Neuroimage [Internet]. 2017;155:177–86. Available from: http://dx.doi.org/10.1016/j.neuroimage.2017.04.068

42. Feng MR, Turluck D, Burleigh J, Lister R, Fan C, Middlebrook A, et al. Brain microdialysis and PK/PD correlation of pregabalin in rats. Eur J Drug Metab Pharmacokinet. 2001;26(1–2):123–8.

43. Deguchi Y, Nozawa K, Yamada S, Yokoyama Y, Kimura R. Quantitative evaluation of brain distribution and blood-brain barrier efflux transport of probenecid in rats by microdialysis: Possible involvement of the monocarboxylic acid transport system. J Pharmacol Exp Ther. 1997;280(2):551–60.

44. Yamamoto Y, Välitalo PA, Huntjens DR, Proost JH, Vermeulen A, Krauwinkel W, et al. Predicting drug concentration-time profiles in multiple CNS compartments using a comprehensive physiologically-based pharmacokinetic model. CPT Pharmacometrics Syst Pharmacol. 2017;6(11):765–77.

45. Yang Z, Brundage RC, Barbhaiya RH, Sawchuk RJ. Microdialysis studies of the distribution of stavudine into the central nervous system in the freely-moving rat. Vol. 14, Pharmaceutical Research. 1997. p. 865–72.

46. Terasaki T, Deguchi Y, Sato H, Hirai K ichi, Tsuji A. In Vivo Transport of a Dynorphin-like Analgesic Peptide, E-2078, Through the Blood–Brain Barrier: An Application of Brain Microdialysis. Vol. 8, Pharmaceutical Research: An Official Journal of the American Association of Pharmaceutical Scientists. 1991. p. 815–20.

47. Arora P, Adams CH, Gudelsky G, DasGupta B, Desai PB. Plasma and brain pharmacokinetics of letrozole and drug interaction studies with temozolomide in NOD-scid gamma mice and sprague dawley rats. Cancer Chemother Pharmacol. 2019 Jan;83(1):81–9.

48. Tsai T, Liang C. Pharmacokinetics of tetramethylpyrazine in rat blood and brain using microdialysis. Int J Pharm. 2001;216:61–6.

49. Kielbasa W, Kalvass JC, Stratford R. Microdialysis evaluation of atomoxetine brain penetration and central nervous system pharmacokinetics in rats. Drug Metab Dispos. 2009 Jan;37(1):137–42.

50. Donovan MD, O’Brien FE, Boylan GB, Cryan JF, Griffin BT. The effect of organic anion transporter 3 inhibitor probenecid on bumetanide levels in the brain: an integrated in vivo microdialysis study in the rat. J Pharm Pharmacol. 2015;67(4):501–10.

51. Chen X, Loryan I, Payan M, Keep RF, Smith DE, Hammarlund-Udenaes M. Effect of transporter inhibition on the distribution of cefadroxil in rat brain. Fluids Barriers CNS. 2014;11(1).

52. Tsai TH. Simultaneous measurement of cefuroxime in rat blood and brain by microdialysis and microbore liquid chromatography Application to pharmacokinetics. J Chromatogr B. 1999;735:25–31.

53. Gynther M, Jalkanen A, Lehtonen M, Forsberg M, Laine K, Ropponen J, et al. Brain uptake of ketoprofen-lysine prodrug in rats. Int J Pharm. 2010;399(1–2):121–8.

54. Xie R, Bouw MR, Hammarlund-Udenaes M. Modelling of the blood-brain barrier transport of morphine-3-glucuronide studied using microdialysis in the rat: Involvement of probenecid-sensitive transport. Br J Pharmacol. 2000;131(8):1784–92.
